# Ultrafast High-Temperature Synthesis of Battery-Grade Graphite Through Energy-Effective Joule Heating: A Combined Experimental and Simulation Study

**DOI:** 10.3390/ma19020348

**Published:** 2026-01-15

**Authors:** Jie-Cong Liu, Qi Li, Salvatore Grasso, Baptiste Py, Zi-Long Wang, Francesco Ciucci, Hua-Tay Lin, Li-Guo Wang, Guang-Lin Nie, Fei Zuo

**Affiliations:** 1 School of Electromechanical Engineering, Guangdong University of Technology, Guangzhou 510006, China; 2 School of Engineering and Materials Science, Queen Mary University of London, London E1 4NS, UK; 3 Department of Mechanical and Aerospace Engineering, The Hong Kong University of Science and Technology, Hong Kong SAR, China; 4 Shenzhen Guochuang New Material Technology Co., Ltd., Shenzhen 518066, China; 5 Guangdong Provincial Key Laboratory of Large Ceramic Plates, Monalisa Group Co., Ltd., Foshan 528211, China

**Keywords:** ultrafast high-temperature graphitization, Joule heating, energy consumption

## Abstract

**Highlights:**

**What are the main findings?**

- A scalable and sustainable ultrafast high-temperature graphitization (UHG) strategy was developed.
- Battery-grade graphite was fabricated within 400 s at a temperature >3000 K.
- UHG products show electrochemical properties comparable to commercial LiB-grade graphite.
- UHG process demonstrates a remarkable reduction in energy consumption.

**What are the implications of the main findings?**

- Technical upgrade of conventional graphite production.
- Rapid synthesis of carbonaceous materials.
- Green upgrading of high-energy-consuming industries.

**Abstract:**

This work introduces ultrafast high-temperature graphitization (UHG) as an effective method to synthesize graphite with significantly reduced processing times of about 100 s and reduced consumed energy, as opposed to conventional methods that require several days at 2800 K. This novel process achieves graphitization of up to 90% within a few minutes due to the accelerated kinetics occurring at temperatures as high as 3400 K. Samples processed using UHG attained stable cyclic capacities of 350 mAh/g, which is fully comparable to commercially available graphite. Finite Element Simulations were also used to calculate the energy consumption for a scaled-up configuration, and it was found that the UHG approach reaches ultra-low energy consumption, requiring only 2.4 MJ/kg for the direct conversion of coke into graphite. By minimizing the duration of high-temperature processing and employing localized heating, UHG is envisioned to mitigate some of the challenges associated with traditional Acheson furnaces that have been in use for more than a century.

## 1. Introduction

Graphite plays a key role as an anode material for Li-ion batteries, where graphite particles are combined with a polymeric binder to ensure good adhesion with a metallic current collector. However, graphite processing is extremely energy-intensive, requiring lengthy exposure to temperatures over 2800 K, depending on both the precursor and the furnace design, for a period of time ranging from five days to two weeks. The graphitization step is still based on the Acheson furnace originally developed in 1896. This process involves a significant amount of electrical energy and is highly polluting, which significantly endangers occupational health [1,2]. In addition to these crucial limitations, Acheson furnaces, which are between 50 and 100 m long, operate in open air and are susceptible to massive and catastrophic explosions whenever air accidentally penetrates isolating bricks and encounters hot graphite. As a result, it is paramount to reduce the heated volumes and favor smaller batch ultrafast processing in order to reduce the severity of these catastrophic accidents.

Manufacturing one ton of synthetic graphite emits between 6 and 12 metric tons of greenhouse gases [1,3]. Natural graphite, due to its scarcity, is already considered a critical material, and its demand is skyrocketing, as a single electric vehicle (EV) requires more than 50 kg [4]. Most of the energy required to produce graphite is consumed during graphitization. Despite being a polluting and scarce resource, little effort has been dedicated to identifying environmentally friendly and more time- and energy-efficient processing routes. Although graphite production itself accounts for less than 0.1% of global emissions [5], EVs could potentially displace 7 teratons of greenhouse gases, which is equivalent to approximately 20% of global emissions, as currently, transportation accounts for 29% of overall emissions. Overall, there is a strong push to efficiently and safely synthesize highly performing graphite anodes using a reduced amount of energy. Recent surveys indicate that graphite shortages for EV production are expected to rise in the coming years, with a projected global supply deficit of 0.8 million tons by 2030 [6]. Due to its energy-intensive manufacturing process, particularly the graphitization step, graphite’s cost has followed recent energy market trends. In this rapidly evolving landscape, graphite emerges as a crucial material set to contribute significantly to the net-zero trajectory driven by the implementation of electric vehicles.

Ultrafast high-temperature heating technology has attracted significant research attention as a potential solution, gaining worldwide recognition and becoming an international academic focus [7]. This technique was originally developed by Wang et al. in 2020, aiming at the consolidation of various structural and functional bulk ceramics [8]. The core concept is to employ a thermally insulating heater energized by the Joule effect. The thermal energy is directed to the workpiece while minimizing heat losses, and it results in a considerably short processing duration, down to a few seconds. A clear benefit is the remarkable energy consumption reduction.

As the heater is typically carbon-based, resulting in minimal cross-contamination, the technique is well suited for processing carbonaceous materials, including graphene and hard carbon derived from different precursors [9,10,11]. Besides time and energy efficiency, ultrafast kinetics allows for tuneable product composition–microstructure–performance relationships, as the process benefits from precise time control [12,13]. However, to the best of the authors’ knowledge, the work on ultrafast high-temperature graphitization (UHG) is very limited in existing literature. With this background, the present work aims to demonstrate the feasibility of UHG for synthesizing graphite within minutes using reduced energy input, which may offer new insights into more sustainable graphitization.

## 2. Materials and Methods

Calcined needle coke (ash content: 0.097 wt.%, moisture content: 0.12 wt.%) was used as the starting precursor. The as-received coke was first heat-treated using a P-UHS system described elsewhere [14]. The precursor to be graphitized was heated at a rate of approximately 750 K/min to temperatures above 3300 K under an argon (Ar) atmosphere. Several preliminary tests were conducted to calibrate the temperature against the melting points of high-purity reference compounds. To control the particle size, the produced graphite was ground and sieved using different mesh sizes. The resulting powders were fabricated into electrode materials and compared with commercial graphite (S_BET_ = 0.7 m^2^/g, D50 = 22.5 μm, Kolod Corporation, Lianyungang, China). Microstructural examination was performed using a field-emission scanning electron microscope (SEM; SU8220, Hitachi, Tokyo, Japan). The degree of graphitization of the samples was evaluated using an X-ray powder diffractometer (XRD; D8 ADVANCE, Bruker, Billerica, MA, USA). A fully automated rapid specific surface area and porosity analyzer (ASAP2460, Micromeritics, Tewkesbury, UK), along with a laser particle size analyzer (Mastersizer 3000, Malvern, Worcestershire, UK), was employed to investigate the effects of specific surface area and particle size. Defect level of the graphite particles was measured using Raman spectroscopy (inVia, Renishaw, Gloucestershire, UK).

The electrode slurry was prepared by thoroughly mixing graphite and Super P at a mass ratio of 9:1, respectively, with polyvinylidene fluoride (PVDF) as the binder and N-methyl-2-pyrrolidone (NMP) as the solvent. The homogeneous mixture was uniformly coated onto a copper foil current collector using a doctor blade coating technique. The coated electrode was vacuum-dried at 90 °C for 12 h to remove residual solvent, followed by precision punching into 12 mm diameter foils. The resulting active material exhibited an areal mass loading of approximately 1.5 mg/cm^2^. CR2032 coin-type cells were assembled in an Ar-filled glovebox with oxygen and moisture levels maintained below 0.1 ppm. The cell configuration consisted of the prepared working electrode, a Li-metal counter electrode, and a Celgard 2500 separator. For the electrolyte solution, we employed 1.0 M lithium hexafluorophosphate (LiPF6) in a mixed solvent of ethylene carbonate (EC) and diethyl carbonate (DEC) (1:1 *v*/*v*). All cell components were meticulously aligned and sealed under controlled pressure to ensure optimal interfacial contact and prevent electrolyte leakage. All batteries were tested using a CT2001A battery testing system (LANHE, Glashütte, Germany).

As discussed thereafter, ultrafast graphitization was observed at extremely high temperatures above 3000 K. As a result, we carried out experimental work using small coke batches of 0.5 g. We validated the simulations against experiments based on 0.5 g batches; however, such a small sample mass was not favorable to reduce the energy consumed during the graphitization. In the second step, we employed simulation for larger coke batches of 3.8 g and 21.1 g of needle coke.

**Figure 1:**
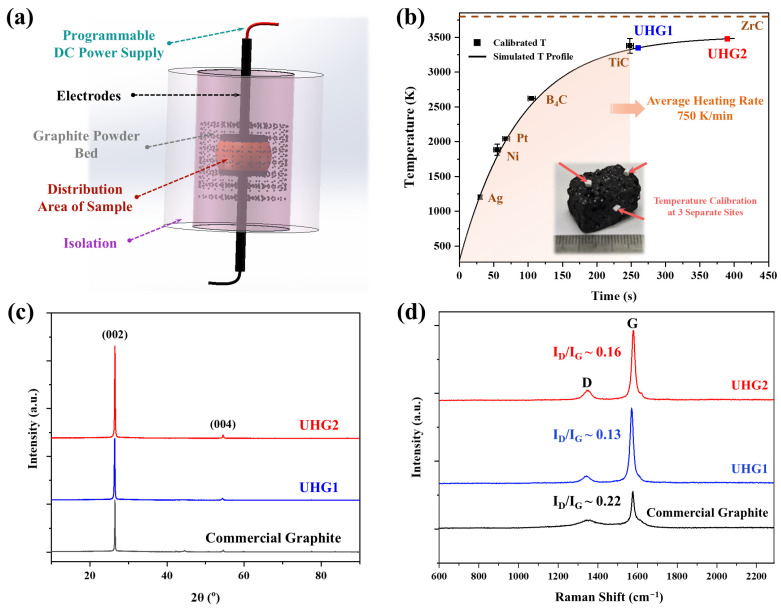
(**a**) Schematics of the UHG system applied in this work. (**b**) Temperature profile of the ultrafast graphitization process. The continuous line represents the temperature simulated by finite element modeling (FEM) [15], while the data points were obtained by melting reference materials with well-defined melting points. For temperature calibration, three small wires/pieces were embedded at different positions on both the top and sides of the sample. The error bars indicate the time interval from partial to complete melting of each wire/piece located at the three probing positions. The reference samples UHG1 and UHG2 have discharge times of 180 s and 300 s at a maximum graphitization current of 95 A, respectively. (**c**) XRD patterns and (**d**) Raman spectra of the corresponding graphitized samples derived from UHG compared to commercial-grade graphite.

Since these experiments would have required an upgraded furnace design, which was beyond the scope of this work, we relied solely on computational modeling to anticipate the reduction in graphitization energy. We further conducted simulations of UHG processes for needle coke of different masses (0.5, 3.8, and 21.1 g) and calculated their energy consumption. Block-shaped samples, as shown in Figure 1b, are suitable for practical experiments and evaluation of graphitization activation energy, while powdered samples were employed for predicting energy consumption in the UHG process, as the coke and the graphite were assumed to be homogenously mixed. In the simulation, the furnace body we designed was a cylindrical shape with a radius and height of 100 mm, while the electrode was a hollow cylindrical structure with an outer diameter of 8 mm and an inner diameter of 5 mm. Among them, the size of the electrode pads increased as the size of the sample mass increased. As for the temperature distribution processing program, we followed the detailed description provided in Reference [15]. In order to achieve more efficient heat transfer between the graphite powder bed and the needle coke sample, different from the experimental stage of work, powdered coke was assumed to be mixed with graphite powder in a 6 to 4 weight ratio, before it was placed in the principal heating area located between two electrode pads. We employed modeling to quantify the energy consumption of UHG in comparison with the existing Acheson process. To do so, the batch based on 3.8 g coke was set to graphitize up to 95% within 1 min, 1 h, and 1 week, respectively.

## 3. Results and Discussion

Figure 1a schematically illustrates the developed UHG setup. The configuration of this device has been described in detail in our previous research [15]. Calcined needle coke was embedded in a graphite powder bed and heated using electrodes connected to a DC power supply. During the electrical discharge, the graphite powder acted as a Joule heating element. Due to the extremely high temperatures involved and the localized heating within the powder bed, direct temperature measurements were not feasible, and the sample temperature was estimated using simulations. Additionally, the temperature data were calibrated using reference materials of well-defined melting points [16]. As shown in Figure 1b, to obtain reliable temperature readings, the heating process was controlled by incrementally increasing the electrical power, and six calibration materials, namely, Ag, Pt, Ni, B_4_C, TiC, and ZrC, were employed. The current was increased at a rate of 1 A per 1 s, corresponding to an average heating rate of 750 K/min. In this study, reference samples UHG1 and UHG2 were processed with applied currents of 95 A and discharge times of 180 s and 300 s, respectively. According to the estimates, the final temperatures of UHG1 and UHG2 exceeded the melting point of TiC (3413 K) but were lower than that of ZrC (3813 K).

Following the UHG process, the obtained samples were first characterized using XRD to investigate the structural parameters. The diffraction peak of the (002) plane, located near 26.5° (d = 0.335 nm), was employed to quantify the degree of graphitization. As shown in Figure 1c, the interlayer spacing of UHG1 (0.3370 nm) decreased to 0.3363 nm for UHG2 with increasing temperature, approaching the value recorded for commercial graphite products (0.3360 nm). The degree of graphitization, DoG, was calculated using the following equation:

(1) $$\mathrm{DoG}\;\left(\%\right)=\frac{0.3440-d\left(002\right)}{0.3440-0.3354}$$

where 0.3440 nm represents the interlayer spacing for turbostratic carbon, which serves as a boundary value between non-graphitized and graphitized carbon for the degree of graphitization calculation, 0.3354 nm is the half-value of the c-axis parameter of the ideal hexagonal graphite crystal, and d(002) is the interlayer spacing of the (002) plane of the sample. The reduction in interlayer spacing is directly related to the increase in DoG. Based on Equation (1), the DoG values for commercial graphite, UHG1, and UHG2 were determined to be 92%, 81%, and 88%, respectively.

For the sake of comparison, conventional synthesis of commercial graphite typically requires hundreds of hours to achieve graphitization with a DoG > 90%. For example, Zhang et al. achieved a degree of graphitization of 78% when dwelling the precursor at 3173 K for 3 h [17]. Similarly, commercial graphite requires a graphitization cycle in the order of 2–3 weeks [18]. In contrast, the UHG graphite synthesized via the UHG process achieves a comparable degree of graphitization within only 400 s, demonstrating significant time efficiency compared to other techniques.

**Figure 2:**
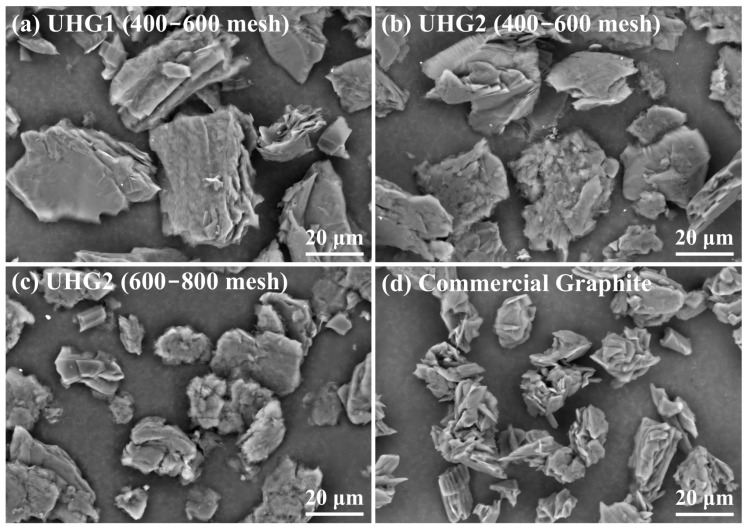
SEM images of the graphite produced using UHG, including (**a**) UHG1 and UHG2 for (**b**) 400–600 mesh and (**c**) 600–800 mesh, and comparison with (**d**) commercial graphite.

Nevertheless, Raman spectroscopy and SEM reveal different microstructural features of carbon products across different graphitization processes. In Raman spectroscopy, the intensity ratio of *D* and *G* peaks (*I_D_*/*I_G_*) implies the defect level in the graphite particles, and the larger the *I_D_*/*I_G_* value, the more defects are present [19]. According to Figure 1d, the weaker intensity rate (0.13~0.16) of UHG graphite should indicate a lower microstructural defect density compared to the commercial-grade graphite (~0.22). The microstructure observations of different UHG samples are shown in Figure 2. The images reveal that the UHG1 sample exhibits a well-defined graphite layer and flake-like morphology (Figure 2a), comparable to the commercial counterparts (Figure 2d). The shorter synthesis time of UHG samples compared to commercial graphite indicates that rapid heating and higher temperature significantly promoted graphitization. However, rough surfaces and crack structures are also visible on the UHG graphite (Figure 2b,c), likely resulting from the combination of short processing time and non-graphitizable carbon structures present in the raw material. Thus, the defect ratio of the graphite particles might be affected by both the synthesis process and post-treatment. A grinding and sieving treatment after the UHG process could also greatly influence the microstructure of graphite particles, especially for the pore/crack size and distribution [20]. These defects contribute to the larger specific surface area of UHG samples compared to commercial graphite, as listed in Table 1, potentially reducing their electrochemical performance. The specific surface area values of UHG1, UHG2 (400–600 mesh), and UHG2 (600–800 mesh) were 2.0, 7.3, and 9.4 m^2^/g, respectively, whereas commercial graphite had a significantly reduced surface area of 0.7 m^2^/g. Particle size distribution analysis was performed using a laser particle size analyzer, and the results are summarized in Table 1. It is evident that the UHG graphite samples have larger particle sizes than commercial grade, a trend also confirmed by the SEM images (Figure 2). Previous studies have reported that an excessively large specific surface area can lead to lower initial coulombic efficiency in electrode materials during electrochemical testing [18].

**Figure 3:**
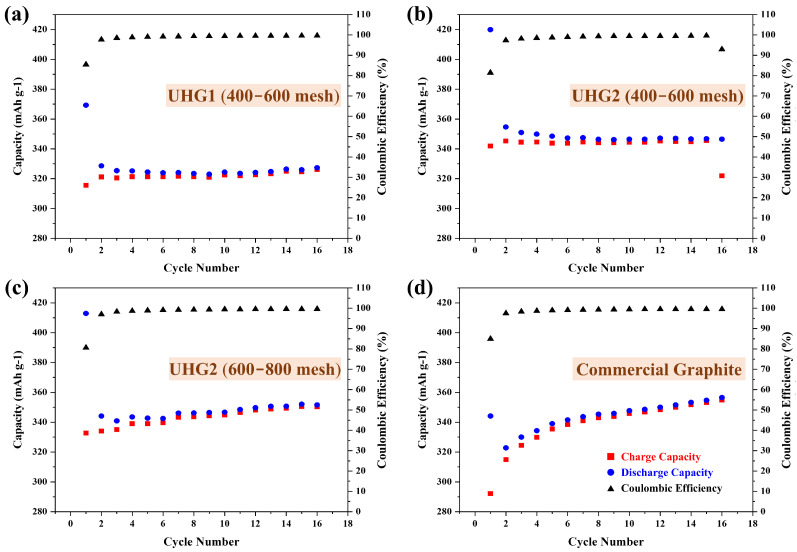
Electrochemical curves at 0.2C rate for (**a**) UHG1 and UHG2, (**b**) 400–600 mesh, and (**c**) 600–800 mesh and compared with (**d**) commercial graphite.

**Figure 4:**
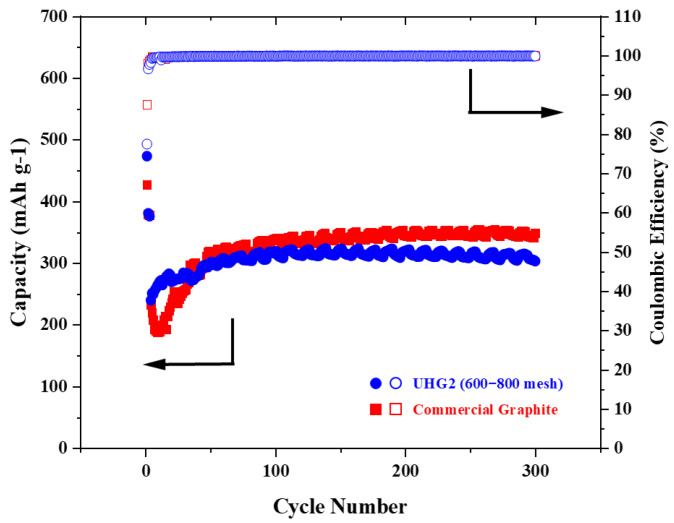
Electrochemical curves at 1C rate for UHG2 (600–800 mesh) and commercial-grade graphite.

Then, the UHG graphite samples were used as electrode materials and compared with electrodes made from commercial graphite at a rate of 0.2C, as shown in Figure 3. Electrochemical data (Table 1) revealed that UHG samples synthesized via the UHG process exhibited higher initial cycle capacities. Notably, the UHG2 sample achieved an initial cycle capacity exceeding 400 mAh/g (Figure 3b,c), significantly higher than the 344 mAh/g observed for commercial graphite (Figure 3d). After the second cycle, the capacities of UHG samples gradually decreased and stabilized above 300 mAh/g. Specifically, the stable capacities were 327 mAh/g for UHG1 (Figure 3a) and 346 mAh/g (Figure 3b) and 350 mAh/g (Figure 3c) for the two UHG2 samples, comparable to the commercial graphite (Figure 3d). Preferably, the UHG2 (600~800 mech) graphite electrode was then chosen and further cycled at the current density of 1C. As shown in Figure 4, relative to the commercial graphite, the UHG one still exhibited higher discharge capacity (473 vs. 426 mAh/g) but lower Coulombic efficiency (78% vs. 87%) at the first cycle, similar to testing under the 0.2C rate. However, UHG graphite maintained 320 mAh/g capacity after 200 cycles (68% retention), while commercial graphite achieved an increased capacity of 345 mAh/g (80% retention).

Electrochemical data demonstrate that the performance of lithium-ion battery electrodes is directly related to the degree of graphitization and structural defect level of the graphite material. According to the current research [21], the higher the degree of graphitization, the closer the efficiency of lithium-ion batteries is to the theoretical value. Compared to UHG1, UHG2 and commercial graphite exhibit higher graphitization degrees, resulting in superior performance (Table 1). Notably, the initial coulombic efficiency of the UHG2 sample is lower than that of commercial graphite. While commercial graphite achieves an initial efficiency of 85% at 0.2C, UHG2 only reaches 81%. This lower efficiency is likely attributed to the larger specific surface area of the UHG samples, which leads to a drop after the first cycle. Previous studies indicate that an excessive specific surface area causes higher lithium-ion consumption during the formation of the solid electrolyte interphase in the first cycle [18]. Once the current rate was increased, the microstructure of UHG graphite with a much higher specific surface area would be subjected to greater impact during the rapid Li-ion intercalation/deintercalation in the cycling process, resulting in a lower capacity retention rate after long cycles.

**Figure 5:**
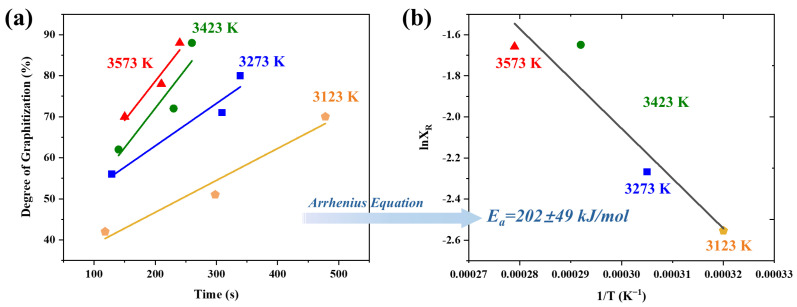
(**a**) Thermodynamic curves of DoG processed within the temperature range of 3123 and 3573 K; (**b**) apparent activation energy of the graphitization process.

The conversion of amorphous carbon into an ordered carbon at such extremely high temperature is not often investigated in the literature. Therefore, to further study the graphitization effect of the UHG process, the DoG was used as a reference to evaluate the kinetic behavior of the P-UHS graphitization process. UHG samples were prepared at different temperatures (3123, 3273, 3423, and 3573 K) under a heating rate of 1000 K/min. To eliminate the effects of temperature nonuniformity, three samples were processed simultaneously in a single UHG run, and the DoG of the samples was estimated based on XRD measurements (see Table 2). It can be seen that the degree of graphitization is a function of both temperature and time. From this, a graphitization–time curve for UHG graphite was constructed in Figure 5a. Additionally, the slope of the graphitization–time curve can be approximately considered as the reaction rate constant during the graphitization process. As expected, the graphitization process reveals that, at a given temperature, the interlayer spacing of carbon materials decreases with increasing temperature and prolonged holding time. This phenomenon indicates that the transition from disordered carbon to ordered carbon structures during graphitization is a highly dynamic process. The kinetic relationship can be described using the following Arrhenius empirical equation:

(2) $$X_{R}\;=\;Ae^{-\mathrm{Ea}/\mathrm{RT}}$$

where *X_R_* represents the reaction rate constant, *A* is the pre-exponential factor, *E_a_* is the apparent activation energy, *R* is the molar gas constant, and *T* is the absolute temperature. Based on Equation (2), a thermodynamic plot was generated using experimental data corresponding to the DoG of the UHG samples. The activation energy was derived from the slope of the plot shown in Figure 5b. The activation energy for the UHG process at a heating rate of 1000 K/min was determined to be 202 ± 49 kJ/mol. As a comparison to the standard Acheson graphitization process, the activation energy for conventional graphitization typically reaches 230 ± 15 kJ/mol [15]. This comparison indicates that the UHG process has comparable apparent graphitization activation energy, confirming that the mechanisms are not changed.

Graphitization is a process in which the atomic migration and structure rearrangement play the major roles. From the thermodynamic viewpoint, the intrinsic driving force for graphitization is the difference in Gibbs free energy between graphite and coke. It depends only on the initial and final states of the thermodynamic parameters describing the overall process, but not on the reaction route. However, to make graphitization happen, a minimum energy must be input to the system in order to jump the potential energy barrier, which prevents atoms from migrating within the structure. This minimum energy is called the “apparent graphitization activation energy”. Relative to the conventional Acheson graphitization route, the UHG process has three remarkably different technical characteristics: fast heating rate, high synthesis temperature, and large current environment. However, an identical apparent activation energy range corresponds to the graphitization mechanisms, which are unaffected by thermal profile and current environment. These findings highlight the potential of the P-UHS system in ultrafast preparation of graphite industry, with estimations provided in Table 2.

**Figure 6:**
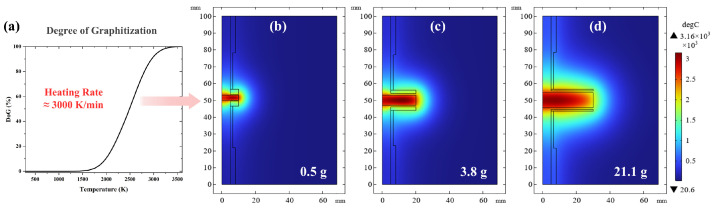
(**a**) Thermodynamic curves showing the degree of graphitization of coke as a function of temperature, assuming a heating rate of 3000 K/min and no dwelling time, and simulation of the resulting temperature distribution for (**b**) 0.5, (**c**) 3.8, and (**d**) 21.1 g batches of coke.

To obtain the relationship between the degree of graphitization time and temperature distribution, we embedded Equation (2) within the finite element software (COMSOL Multiphysics 6.2) (Figure 6a). Setting as a target a DoG of 95%, the corresponding temperature distribution for different coke loading are shown in Figure 6b–d. As can be seen from Table 3, energy consumption decreases as the sample mass increases. From the simulation, it can also be concluded that the UHG process has lower energy consumption because of the reduced losses associated with the larger volume-to-surface ratio of the heated graphitization zone.

**Figure 7:**
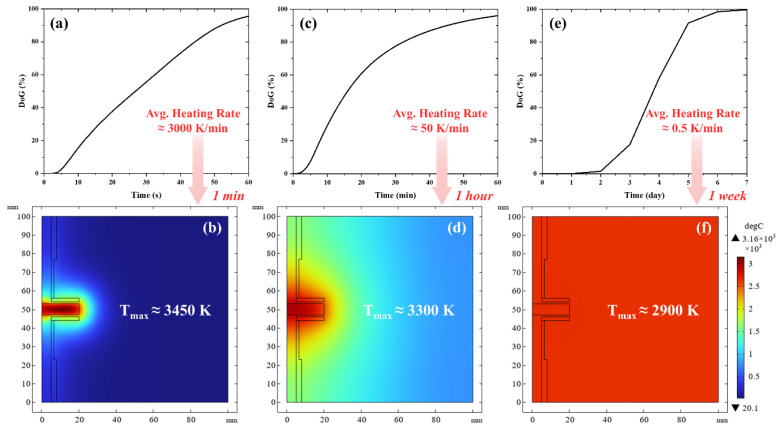
Process curve of the sample reaching a 95% degree of graphitization within different time intervals and the corresponding simulated temperature distribution of the samples reaching 95% graphitization: (**a**,**b**) 1 min, (**c**,**d**) 1 h, and (**e**,**f**) 1 week. Note that the even temperature distribution in f is due to the absence of heat losses.

To further elucidate the relationship between energy consumption and processing kinetics, additional simulations were conducted to investigate the time duration dependency of the graphitization process. Heating simulations were performed for the 3.8 g batch under three distinct time scales, setting as a target a 95% graphitization within 1 min, 1 h, and 1 week, roughly matching the Acheson process. The simulation plots the corresponding temperature distribution in Figure 7, and the corresponding energy consumption is presented in Table 3, which reveals a clear inverse relationship between heating rate and energy efficiency. Conventional graphite production methods demonstrate significantly higher energy consumption. For instance, the traditional Acheson process requires approximately 10.8–28.8 MJ/kg for synthetic graphite production according to industrial data, with some studies reporting energy consumption as high as 66.2 MJ/kg, due to the marked difference in expended electrical energy [2,18]. In direct contrast, the UHG process achieves a remarkably low energy consumption of 4.5 MJ/kg with a minute duration processing time, representing up to 85% reduction compared to conventional methods.

This dramatic energy efficiency advantage is derived from minimized thermal losses during rapid UHG heating [2]. The graphitization furnace typically operates at temperatures over 2800 K, contributing to the high energy intensity of conventional processes [22]. The UHG process not only achieves superior time efficiency but also demonstrates exceptional energy optimization potential. These findings suggest that UHG technology could potentially reduce the carbon footprint associated with synthetic graphite production, addressing both economic and environmental concerns critical for sustainable battery materials manufacturing.

## 4. Conclusions

In this work, ultrafast high-temperature graphitization (UHG) graphite was developed. A combined experimental and modeling study was conducted to correlate processing with particle size, surface area, and electrochemical performance. Experimental results based on XRD analysis revealed that UHG graphite synthesized with a heating rate of 750 K/min reached 88% graphitization within 400 s, representing an unprecedented time reduction in the synthesis compared to conventional techniques. Furthermore, electrochemical performance analysis revealed that UHG graphite exhibits charge–discharge characteristics with well-developed graphitic crystallinity and minimal defect density comparable to commercial-grade graphite.

Using modeling data, the UHG process demonstrates remarkable energy efficiency, consuming only 2.4 MJ/kg for a 21.1 g coke batch size compared to conventional methods requiring more than 10.8 MJ/kg. This represents at least a 60% reduction in energy consumption through minimized thermal losses of the thermally insulating powder bed and elimination of prolonged high-temperature exposure. Simulation calculations confirm that energy efficiency scaled favorably with batch size, and shorter processing times consistently yield lower energy consumption.

Despite the promising results, additional research is required to fully understand the UHG graphitization mechanisms, and further efforts are required for the scaling-up considerations and applicability of the methodology when operating at such ultrahigh temperatures. In principle, the UHG technology offers a promising pathway to address both high energy consumption in conventional graphite production and the environmental impact associated with Li battery anode graphitization.

## Figures and Tables

**Table 1:** Comparative analysis between UHG and commercial graphite, including the particle size of the samples measured using laser diffraction, the BET surface area, the degree of graphitization, and the electrochemical anode performance measured at the rate of 0.2C.

Sample ID	Avg. Particle Size (μm)	BET Surface Area (m^2^/g)	Avg. DoG (%)	Electrochemical Test (at 0.2C)		
				First Cycle Discharge Capacity (mAh/g)	First Cycle Efficiency (%)	Stable Capacity After 20 Cycles (mAh/g)
**UHG1** **(400–600 mesh)**	26.8	2.0	81.1 ± 1.1	369	86	327
**UHG2** **(400–600 mesh)**	29.4	7.3	88.4 ± 0.5	420	81	346
**UHG2** **(600–800 mesh)**	20.4	9.4	88.4 ± 0.5	413	81	350
**Commercial Graphite**	22.5	0.7	92.1 ± 0.2	344	85	356

**Table 2:** Estimation of the degree of graphitization obtained at 3123–3573 K for different dwell times, under a heating rate of 1000 K/min.

Temperature (K)	Time (s)	Avg. DoG (%)	Temperature (K)	Time (s)	Avg. DoG (%)
**3123**	0	42	**3423**	0	62
	180	51		90	72
	360	70		120	88
**3273**	0	56	**3573**	0	70
	180	71		60	78
	210	80		90	88

**Table 3:** Gravimetric energy consumption of needle coke for different batch weights processed under different heating rates and dwelling time of 30 s, targeting a DoG of 95%.

**Sample Mass (g)**	0.5	3.8	21.1	3.8		
**Avg. Heating Rate (K/min)**	3000			3000	50	0.5
**Unit Energy** **Consumption (MJ/kg)**	8.6	4.5	2.4	4.5	48.0	101.1

## Data Availability

The original contributions presented in this study are included in the article. Further inquiries can be directed to the corresponding authors.
